# The pharmacokinetic profile of brensocatib and its effect on pharmacodynamic biomarkers including NE, PR3, and CatG in various rodent species

**DOI:** 10.3389/fphar.2023.1208780

**Published:** 2023-07-19

**Authors:** Jessica Basso, Kuan-Ju Chen, Yuchen Zhou, Lilly Mark, Daniel LaSala, Arielle Dorfman, Mary Atalla, Donald Chun, Veronica Viramontes, Christina Chang, Franziska Leifer, Patrick P. McDonald, David C. Cipolla

**Affiliations:** Insmed Incorporated, Bridgewater, NJ, United States

**Keywords:** neutrophil elastase (NE), proteinase 3 (PR3), cathepsin G (CatG), cathepsin C (CatC), dipeptidyl peptidase 1 (DPP1), brensocatib

## Abstract

Brensocatib is a novel, oral, selective, reversible inhibitor of dipeptidyl peptidase 1 (DPP1), which activates several neutrophil serine proteases (NSPs), including neutrophil elastase (NE), proteinase 3 (PR3), and cathepsin G (CatG) in the bone marrow during the early stage of neutrophil maturation. These NSPs are associated with pathogen destruction and inflammatory mediation; their dysregulated activation can result in excess secretion of active NSPs causing damaging inflammation and contributing to neutrophil-mediated inflammatory and autoimmune diseases. Pharmacological inhibition of DPP1 in the bone marrow could therefore represent an attractive strategy for these neutrophil-driven diseases. A completed Phase 2 trial in non-cystic fibrosis bronchiectasis patients (ClinicalTrials.gov number NCT03218917; EudraCT number: 2017-002533-32) indeed demonstrated that administration of brensocatib attenuated the damaging effects of chronic inflammation by inhibiting the downstream activation of NSPs. To support a range of preclinical programs and further understand how rodent species and strains may affect brensocatib’s pharmacokinetic (PK) profile and its pharmacodynamic (PD) effects on NE, PR3, and CatG, an extensive naïve dosing study with brensocatib at different dosing levels, frequencies, and durations was undertaken. Dose-dependent PK exposure responses (AUC and Cmax) were observed regardless of the rodent species and strain. Overall, mice showed greater reduction in NSP activities compared to rats. Both mice and rats dosed once daily (QD) had equivalent NSP activity reduction compared to BID (twice a day) dosing when the QD dose was 1.5-times the BID daily dose. For both mouse strains, CatG activity was reduced the most, followed by NE, then PR3; whereas, for both rat strains, PR3 activity was reduced the most, followed by CatG, and then NE. Maximum reduction in NSP activities was observed after ∼7 days and recoveries were nearly symmetrical. These results may facilitate future *in vivo* brensocatib study dosing considerations, such as the timing of prophylactic or therapeutic administration, choice of species, dosage and dosing frequency.

## 1 Introduction

Neutrophil elastase (NE), proteinase 3 (PR3), and cathepsin G (CatG) are structurally related enzymes that are stored within the neutrophil cytoplasmic azurophilic granules and are collectively known as neutrophil serine proteases (NSPs) (Pham, 2008; Korkmaz et al., 2010). They are synthesized as inactive pre-pro-proteins during granulocyte development and are converted to active proteins by dipeptidyl peptidase 1 (DPP1, also known as cathepsin C) during neutrophil maturation in the bone marrow (Turk et al., 2001; Stapels et al., 2015; Palmer et al., 2018). NSPs play a foremost role in pathogen destruction, contributing to inflammatory regulation and modulation, and are known to operate in at least three manners (Pham, 2008): during phagocytosis when the granules fuse with the phagosome (Korkmaz et al., 2010); upon neutrophil activation when granule contents can be released into the extracellular milieu; and (Stapels et al., 2015) upon extrusion of neutrophil extracellular traps (NET) that are comprised of chromatin decorated with histones and proteases (Pham, 2006; Pham, 2008; Kaplan and Radic, 2012; Stapels et al., 2015). Of concern, however, is that dysregulated neutrophil activation can result in excess secretion of active NSPs, thereby contributing to damaging inflammation and tissue matrix destruction in several neutrophil-mediated inflammatory diseases (Almansa et al., 2012; Chalmers et al., 2017; Dai et al., 2017; Dittrich et al., 2018; Fresneda Alarcon et al., 2021).

Brensocatib is a novel, small molecule, competitive, oral, selective, reversible DPP1 inhibitor. It is currently in a Phase 3 clinical trial (ClinicalTrials.gov number NCT04594369) in non-cystic fibrosis bronchiectasis (NCFBE) and its pharmacological mechanism of action is to inhibit DPP1, and thereby NSP activation in neutrophils, but has no direct inhibitory effect on the active NSPs themselves. In a completed Phase 2 trial in NCFBE (the WILLOW study, NCT03218917), brensocatib demonstrated a dose-dependent reduction in sputum NE activity and prolonged time to first exacerbation compared to placebo (Chalmers et al., 2020). Dose-dependent reductions in sputum PR3 and CatG activities were likewise observed (Cipolla et al., 2023). Thus, inhibiting DPP1 in diseases where neutrophils are present and highly activated is likely to attenuate tissue damage that results from the excessive release of active NSPs.

Continued preclinical research efforts are warranted to understand the impact of inhibiting DPP1, and thus reducing the formation of active NSPs, on other chronic diseases characterized by neutrophilic inflammation and tissue damage. Mouse and rat preclinical models are the most frequently used *in vivo* models for drug discovery and development (Hickman et al., 2017) due to various advantages such as their small size, ease of maintenance, they frequently display similar biological pathways to humans, and the possibility to investigate all clinically relevant routes of administration (Turner et al., 2011; Bryda, 2013; Hickman et al., 2017). Importantly, there is an abundance of strains to choose from including inbred, outbred, transgenic, knockout, and congenic (Bryda, 2013; Hickman et al., 2017). Choice of species, strain, and background is important since it can influence disease phenotype, progression, and severity (Doetschman, 2009; Gueders et al., 2009; Livraghi-Butrico et al., 2012; Walkin et al., 2013).

To support a range of preclinical studies, an extensive naïve dosing study was undertaken in each of the two most common mouse and rat research strains and backgrounds, i.e., C57BL/6 and BALB/c mice, and Sprague Dawley (SD) and Wistar rats. The objective was to define brensocatib’s pharmacokinetic (PK) profile and pharmacodynamic (PD) effect on NE, PR3, and CatG when administered orally at different dosing levels, frequencies, and durations.

## 2 Materials and methods

### 2.1 Animals

All studies were carried out under the guidance of the Institutional Animal Care and Use Committee (IACUC) of Rutgers University Animal Care Committee (Protocol No: PROTO999900342). Mice were purchased from Jackson Laboratories, Bar Harbor, ME and rats were purchased from Charles River Laboratories, Wilmington, MA. Animals were between the ages of 8–9 weeks at the start of each study. At the conclusion of each study, animals were sacrificed via CO2 inhalation followed by cardiac bleed.

### 2.2 Dosing

For each study, brensocatib was formulated in a solution of 0.5% hydroxypropyl methylcellulose (HPMC) in sodium citrate buffer with 0.1% Tween 80, pH 3.0 for oral dosing.

Oral dosing was performed *via* single use plastic oral gavage feeding tubes from Instech that were appropriately sized for the animal to avoid trauma to internal structures.

### 2.3 BID vs QD dosing study in mice and rats

A dose frequency study was performed in male and female mice (C57BL/6 & BALB/c) and in male and female rats (Sprague Dawley & Wistar) investigating twice daily (BID) *versus* once daily (QD) brensocatib administration.

Briefly, animals were weighed and randomized into different treatment groups. In total, three different dosing levels of brensocatib were tested in each species and strain; each QD dosing level was equivalent to 1.5-times the matching daily BID dose. For BID dosing, the second daily dose was administered 8-h post the morning dose. C57BL/6 mice were dosed either BID or QD for 7 consecutive days at 0/0, 0.5/0.75, 2/3, and 20/30 mg/kg/day (BID/QD). BALB/c mice were dosed for 7 consecutive days at 0/0, 0.2/0.3, 2/3, and 20/30 mg/kg/day (BID/QD). SD rats were dosed for 9 consecutive days at 0/0, 0.2/0.3, 2/3, and 20/30 mg/kg/day (BID/QD). Wistar rats were dosed for 7 consecutive days at 0/0, 0.2/0.3, 2/3, and 20/30 mg/kg/day (BID/QD).

For sparse PK analysis *via* LC-MS/MS, plasma was collected from animals during the first 24 h of dosing at prescribed timepoints. The timepoints included 1, 3, 8, and 24 h post initial dose for QD groups, and 1, 3, 8, 9, 11, and 24 h post initial dose for BID groups. Note that the 8- and 24-h plasma samples were prior to the afternoon dose for BID groups and prior to the second day dose for all groups, respectively. See section *Quantification of Plasma Brensocatib Concentrations* for details.

At the completion of the study, animals were sacrificed 16–24 h post their final dose. The hind limb bones (femurs and tibias) were collected at necropsy for bone marrow analysis of NSP activities by kinetic enzymatic assays (Basso et al., 2022). See section *Quantification of Bone Marrow NSP Activities* for details.

### 2.4 PD onset dosing study in C57BL/6 mice

A 14-day PD onset study was performed in female C57BL/6 mice dosed BID. Mice were dosed by oral gavage either with vehicle (0 mg/kg) or 5 mg/kg brensocatib (BID; 10 mg/kg/day) for 1, 4, 7, 10, or 14 consecutive days. Plasma was collected from animals during the first 24 h of dosing at 1, 2, 8, 9, 10, and 24 h post the initial dose. Note the 8- and 24-h plasma samples were prior to the afternoon dose and the second day dose (if applicable), respectively. Plasma was used for PK analysis via LC-MS/MS. On prescribed days, animals were sacrificed 16 h post final dose, and the hind bones (femurs and tibias) were collected at necropsy for bone marrow analysis of NSP activities by kinetic enzymatic assays.

### 2.5 PD recovery dosing study in C57BL/6 mice

A PD recovery study was performed in female C57BL/6 mice dosed BID for 8 consecutive days. Mice were dosed by oral gavage either with vehicle (0 mg/kg) or 5 mg/kg brensocatib (BID; 10 mg/kg/day). After 8 consecutive days of dosing, the animals were maintained without further treatment until prescribed sacrifice (days: 1, 3, 6, 8, and 10 days post final dose). Plasma was collected from animals during the first 24 h of dosing at −0.5 (half-hour prior to dosing), 1, 8, 9, and 24 h post initial dose. Note that the 8- and 24-h plasma samples were prior to the afternoon dose and prior to the second day dose, respectively. Plasma was used for PK analysis via LC-MS/MS. On prescribed days, animals were sacrificed, and the hind limb bones (femurs and tibias) were collected at necropsy for bone marrow analysis of NSP activities by kinetic enzymatic assays.

### 2.6 Blood and bone marrow collection

Blood was collected via the tail-vein technique and centrifuged to separate and collect the plasma. The resulting plasma samples were stored at −20°C or lower until LC-MS/MS analysis.

For the mice, each animal had the femurs and tibias of both hind limbs harvested. For the rat, the femur and tibia from a single hind limb per animal was harvested. Bones were kept on wet ice until processing for bone marrow collection. In brief, bone cutters were used to cut the epiphysis (knobby ends) off the long bones to expose the bone marrow filled cavity. Each bone cavity was then flushed with 5 mL/bone (mouse) or 10 mL/bone (rat) of ice-cold RPMI media through a 40 μM cell strainer and collected in a 50-mL conical tube. Cells were spun down at 2,500 × g at 4°C for 5 min and the supernatant was aspirated. After which, bone marrow cells were lysed with 1X red blood cell (RBC) lysis buffer (Abcam, ab204733) to eliminate contaminating RBCs and washed with PBS to remove any residual RBC lysis buffer. The resulting white blood cells (WBC) were subsequently lysed with 1% (v/v) Triton X-100 in PBS. Lysates were added to 96-well plates and stored at −80°C until NSP analyses.

### 2.7 Quantification of Plasma Brensocatib Concentrations

The plasma concentration of brensocatib was quantified by liquid chromatography, tandem mass spectrometry (LC-MS/MS), a Sciex API6500 mass spectrometer (SCIEX, Framingham, MA). Two brensocatib stock solutions were prepared, one for the preparation of the 8-point standard (STD) curve spiking solutions and the other for the preparation of the quantitative control spiking solutions (QCs) at three levels. The STD curve and QCs were prepared by 2% spiking from the spiking solutions into mouse or rat plasma. The samples were extracted using a protein precipitation method, where 10 µL of sample was mixed with 100 µL of internal standard working solution (100 ng/mL ^6^C13-brensocatib in methanol), vortexed for 5 s, and centrifuged at 21,130 × g at 4°C for 15 min. The supernatant was mixed with an equal volume of water and 10 μL was injected into a Phenomenex Kinetex Biphenyl column (3.0 mm × 50 mm; 2.6 μm; 65°C; 0.8 mL/min) with an elution gradient consisting of mobile phase A (10 mM ammonium formate) and mobile phase B (methanol) over 2 min from 5% to 95% B. The mass spectrometer was equipped with an atmospheric-pressure chemical ionization source operating in positive mode.

### 2.8 Pharmacokinetic (PK) analysis

Individual plasma concentrations were plotted and the area under the curve (AUC) was calculated using the trapezoid method. The elimination rate constant (*k*_*e*_) was calculated as the negative natural log of the plasma concentration over time (Eq. 1) starting at the max concentration (*C*_max_) after the most recent dose to the last measurable plasma concentration between doses. The calculated *k*_*e*_ was used to define the half-life (*t*_*1/2*_) based on Eq. 2; where 0.693 is the natural log of 2 and assumes a first-order reaction.

$$k_{e}=-\ln\frac{∆C}{∆t}$$ (1)

$$t_{1/2}=\frac{0.693}{k_{e}}$$ (2)

### 2.9 Quantification of Bone Marrow NSP activities

Bone marrow lysates prepared as described above were diluted and plated onto 96-well assay plates to quantify NSP activities as described previously (Basso et al., 2022). Briefly, peptide substrates for NE (N-Methoxysuccinyl-Ala-Ala-Pro-Val-7-amido-4-methylcoumarin; Sigma; St. Louis, MO; Excitation/Emission at 350/450 nm), PR3 ((7-Methoxycoumarin-4-yl)acetyl-lysyl-(picolinoyl)-Tyr-Asp-Ala-Lys-Gly-Asp-N-3-(2-4-dinitrophenyl)-2-3-diaminopropyonyl-NH2); GenScript; Piscataway, NJ; Excitation/Emission at 340/430 nm), and CatG (N-Succinyl-Ala-Ala-Pro-Phe p nitroanilide; Sigma; Absorbance at 405 nm) were added at a final assay concentration of 100 μM, 40 μM, and 200 μM, respectively, and either fluorescence or absorbance was quantified using a Synergy microplate reader (BioTek; Winooski, VT). The specific NSP activity in each sample was calculated as the total activity minus the activity in the presence of a specific NSP inhibitor (if used), including elastase inhibitor (Abcam) for NE, sivelestat (Abcam) for PR3, and cathepsin G inhibitor I (Cayman Chemical; Ann Arbor, MI) for CatG. NSP concentrations were interpolated based on their activities relative to the standard curves created using active human NE protein (Sigma), active human PR3 protein (Sigma), and active human CatG protein (Sigma), respectively. Due to unavailability of commercial mouse and/or rat NE, PR3, and CatG proteins, the corresponding human proteins were used instead since their catalytic ability is considered to be conserved across species. A portion of each cell lysate sample was retained for protein quantification using a Pierce^™^ BCA Protein Assay Kit (Thermo Fisher). The NSP activities were normalized for the cell lysate protein concentrations.

### 2.10 Total protein normalization

The Pierce^™^ BCA Protein Assay Kit was used to measure the total protein of the samples after lysis to allow for subsequent normalization. The manufacturer’s instructions for use were followed when performing this assay on a portion of the sample lysates which were diluted in PBS, the same diluent as used for preparation of the standard. Plates were read on the Biotek Synergy plate reader with Gen5 software.

### 2.11 Statistical analysis

Unless otherwise stated, data are represented as the mean ± SEM. Outliers were determined and excluded *via* Grubb’s Outlier test. All statistical analyses were performed as indicated in the figure legends using Prism 9 software (GraphPad Software, San Diego, CA, United States).

## 3 Results

### 3.1 BID *versus* QD dosing

A pilot study investigating equivalent daily dosing of brensocatib when administered as BID or QD dosing frequencies observed a greater PD biomarker response for the BID administration profile (data not shown). Therefore, to achieve similar PD efficacy between BID and QD studies, the QD dose was increased to 1.5-fold the chosen BID dose. Four dosing studies were then conducted investigating three dosage levels and two dosing frequencies in two mouse strains and two rat strains.

PK parameters including area under curve (AUC) and half-life (t_½_) were calculated as part of PK investigations. To understand brensocatib’s PD effect on biomarkers relevant to the drug’s mechanism of action, NE, PR3, and CatG were measured in lysates generated from hind limb bone marrow collections at sacrifice.

#### 3.1.1 PK: C57BL/6 and BALB/c mice

**FIGURE 1:**
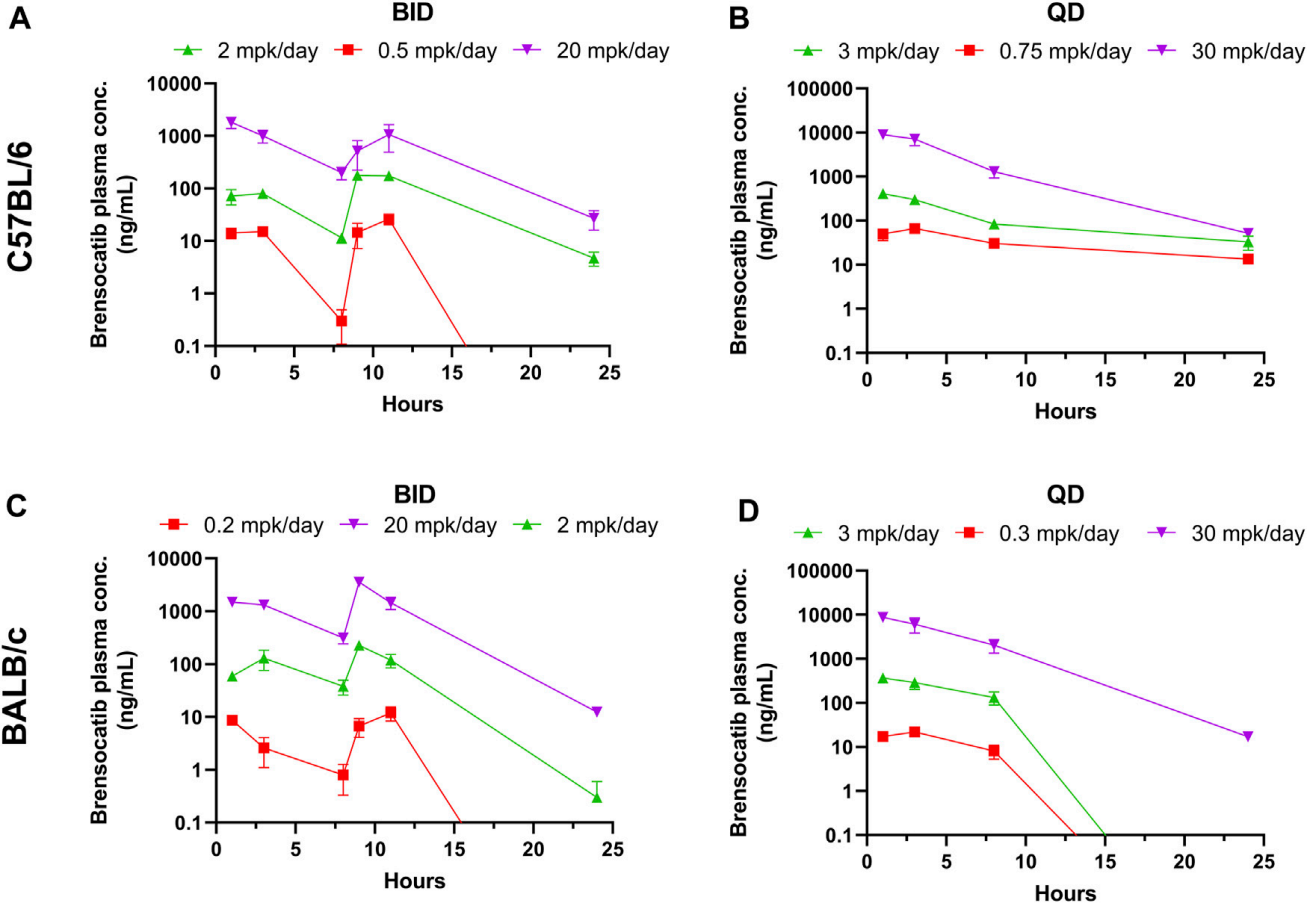
Brensocatib Plasma Concentrations from Repeated Dose Administration of Brensocatib by Oral Gavage. **(A)** BID in C57BL/6 mice, **(B)** QD in C57BL/6 mice, **(C)** BID in BALB/c mice **(D)** QD in BALB/c mice. Data are plotted as mean ± SEM (*n* = 4, except for 11 h of 20 mg/kg BID in C57 where *n* = 3; 3 mg/kg QD in C57 for all time points where *n* = 6; 30 mg/kg QD in C57 for all time points where *n* = 6; and 1 h of 2 mg/kg BID in BALB/c where *n* = 3). mpk/day = mg/kg/day.

Brensocatib was administered orally to C57BL/6 mice for 7 consecutive days with both BID and QD dosing. The brensocatib plasma levels during the first 24 h of administration are shown in Figure 1, and PK parameters are listed in Table 1.

**TABLE 1:** PK parameters of brensocatib in C57BL/6 and BALB/c mice during the first 24 h of BID or QD dosing.

		BID			QD		
C57BL/6	Dose (mg/kg/day)	0.5	2	20	0.75	3	30
	AUC_0–24h_ (ng•h/mL)	294 ± 49	2012 ± 154	15,866 ± 3505	735 ± 108	2843 ± 302	52,565 ± 6720
	AUC_(0–24)_ (µmol•h/L)	0.70 ± 0.12	4.79 ± 0.37	37.73 ± 8.33	1.75 ± 0.26	6.76 ± 0.72	125.01 ± 15.98
	t_1/2_ (h), AM dose	0.71 ± 0.23	2.07 ± 0.42	2.41 ± 0.42	9.62 ± 0.95	6.38 ± 0.56	3.01 ± 0.18
	t_1/2_ (h), PM dose	0.63 ± 0.01	2.35 ± 0.35	2.36 ± 0.05	n/a	n/a	n/a
BALB/c	Dose (mg/kg/day)	0.2	2	20	0.3	3	30
	AUC_0–24h_ (ng•h/mL)	124 ± 31	1508 ± 395	24,105 ± 4779	188 ± 31	2964 ± 679	56,303 ± 17,303
	AUC_(0–24)_ (µmol•h/L)	0.29 ± 0.07	3.59 ± 0.94	57.32 ± 11.36	0.45 ± 0.07	7.05 ± 1.61	133.90 ± 41.15
	t_1/2_ (h), AM dose	1.58 ± 0.72	3.22 ± 0.35	2.47 ± 0.31	0.97 ± 0.03	0.83 ± 0.03	2.56 ± 0.14
	t_1/2_ (h), PM dose	0.67 ± 0.04	1.19 ± 0.34	1.88 ± 0.06	n/a	n/a	n/a

AUC, area under the concentration-time curve; t_1/2_ = half life. Data are presented as mean ± SEM (n = 4, except for C57 20 mg/kg/day where n = 3, C57 3 mg/kg/day and 30 mg/kg/day where n = 6, and Balb/c 2 mg/kg/day where n = 3). n/a, not applicable.

Both dosing regimens showed a dose-dependent trend of plasma exposure (Figure 1A, B). The plasma AUC_0–24h_ in BID dosed mice was 0.70, 4.79, and 37.73 μmol h/L for the 0.5, 2, and 20 mg/kg/day doses, respectively. The plasma AUC_0–24h_ in QD dosed mice was 1.75, 6.76, and 125.01 μmol h/L for the 0.75, 3, and 30 mg/kg/day doses, respectively (Table 1). The slightly higher AUC for the QD animals can be attributed to the 1.5-fold greater daily dose exposure to brensocatib, and in fact the QD AUC ranged between 1.4- and 3.3-fold higher than the BID AUC for the respective doses.

The t_½_ for BID dosing in the morning was 0.71, 2.07, and 2.41 h for the 0.5, 2, and 20 mg/kg/day doses, respectively and were comparable to the evening dose t_½_, which were 0.63, 2.35, and 2.36 h. The t_½_ for QD dosing was 9.62, 6.38, and 3.01 h for the 0.75, 3, and 30 mg/kg/day doses, respectively (Table 1). Both dosing regimens showed a relatively short half-life of brensocatib.

The same dosing study was repeated in BALB/c mice. Unlike with the C57BL/6 mice, signs of aggressive and/or barbering behavior were observed during the study in the BALB/c mice. To ensure the health and safety of the animals, animals exhibiting signs of aggression were placed in separate cages to prevent further behavior. The animals that exhibited this behavior were in the following groups and cages: two male animals in the 0.2 mg/kg/day BID group that were originally caged with BID vehicle animals, and two male animals in the 30 mg/kg/day QD group and one male animal in the 3 mg/kg/day QD that were originally caged together. This aggressive behavior is commonly seen in this mouse strain and sex.

The brensocatib plasma levels during the first 24 h of administration are shown in Figure 1, and PK parameters are listed in Table 1. For BALB/c mice, both dosing regimens showed a dose-dependent trend of plasma exposure (Figures 1C, D). The plasma AUC_0–24h_ in BID dosed mice was 0.29, 3.59, and 57.32 μmol h/L for the 0.2, 2, and 20 mg/kg/day doses, respectively. The plasma AUC_0–24h_ in QD dosed mice was 0.45, 7.04, and 133.90 μmol h/L for the 0.3, 3, and 30 mg/kg/day doses, respectively (Table 1). A slightly higher AUC for the QD animals due to the greater daily dose exposure to brensocatib was also observed in this strain and the ratio of QD to BID AUC ranged from 1.6 to 2.3 for the respective doses.

The t_½_ for BID dosing was 1.58, 3.22, and 2.47 h for the 0.2, 2, and 20 mg/kg/day doses, respectively, in the morning *versus* 0.67, 1.19, and 1.88 h in the evening for those doses. The t_½_ for QD dosing was 0.97, 0.83, and 2.56 h for the 0.3, 3, and 30 mg/kg/day doses, respectively (Table 1). Similar to C57BL/6, a relatively short half-life of brensocatib was observed in BALB/c mice in both dosing regimens.

#### 3.1.2 PD: C57BL/6 and BALB/c mice

**FIGURE 2:**
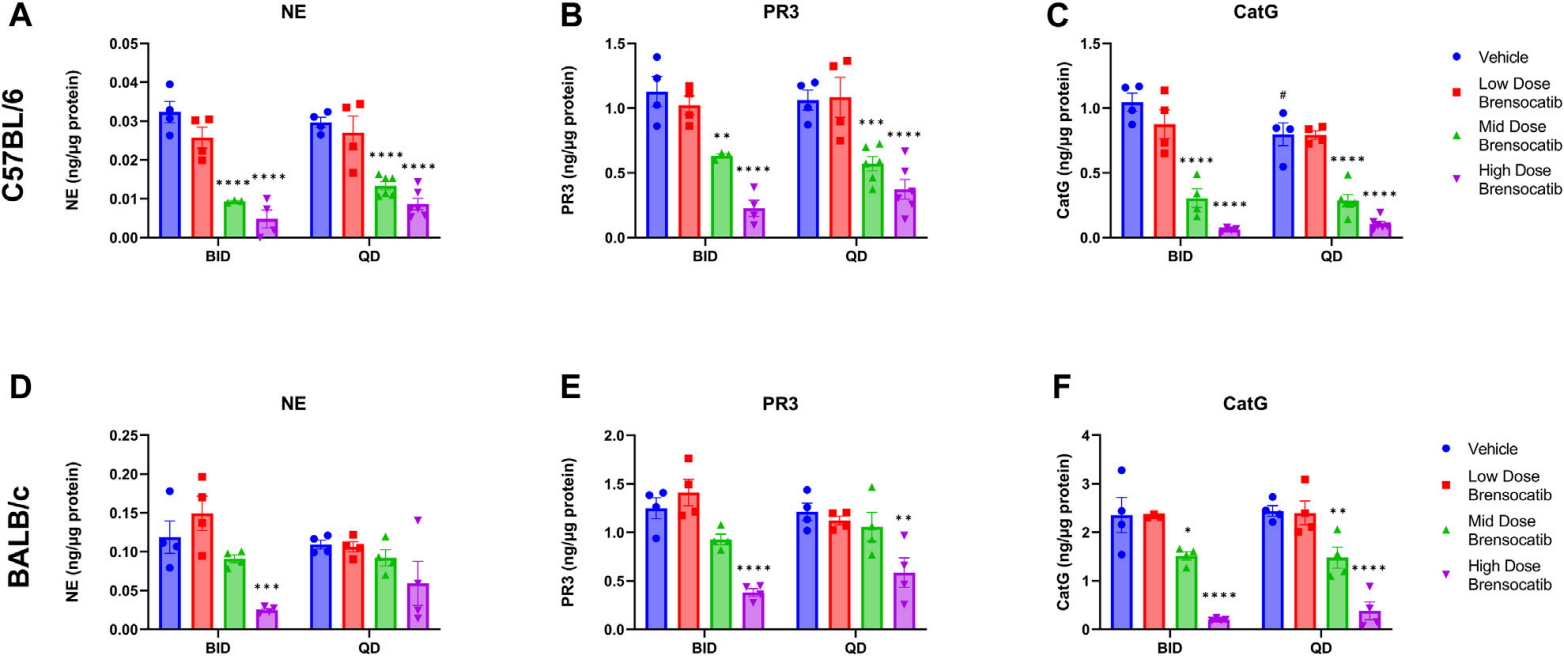
NSP Activities in Bone Marrow of **(A–C)** C57BL/6 and **(D–F)** BALB/c Mice Administered 7-Day Repeated Doses of Brensocatib. Reported NSP activities are 16 and 24 h post final BID and QD dose, respectively. Data are the plotted as mean ± SEM (*n* = 4, except for mid and high C57 QD groups where *n* = 6; and C57 NE and PR3 mid-dose BID and CatG low dose brensocatib BID where *n* = 3 due to outlier which was determined by Grubb’s test). *, *p* < 0.05 vs. vehicle; **, *p* < 0.01 vs. vehicle; ***, *p* < 0.001 vs. vehicle; ****, *p* < 0.0001 vs. vehicle; #, *p* < 0.05 vs. BID-QD.

The bone marrow NSP activity levels after the 7-day repeated doses of brensocatib in C57BL/6 and BALB/c mice are shown in Figure 2 for both BID and QD dosing regimens.

For the C57BL/6 mice, when compared to the appropriate vehicle control, a dose-dependent reduction was observed in bone marrow for all three NSP activities. BID dosing at the highest dose reduced NE, PR3, and CatG by 85% (*p* < 0.0001), 80% (*p* < 0.0001), and 94% (*p* < 0.0001), respectively. QD dosing at the highest dose reduced NE, PR3, and CatG activities by 71% (*p* < 0.0001), 65% (*p* < 0.0001), and 87% (*p* < 0.0001), respectively (Table 2).

**TABLE 2:** Brensocatib treatment percentage change from vehicle control for BID and QD dosing in C57BL/6 and BALB/c mice. *p*-values derived from Sidak’s multiple comparison test.

			*Change From Vehicle Control (%)*		
			NE	PR3	CatG
*C57BL/6*	BID	0.5 mg/kg/day Brensocatib	−20% (*p* = 0.1494)	9% (*p* = 0.8059)	−16% (*p* = 0.1964)
		2 mg/kg/day Brensocatib	−71% (*p* < 0.0001)	−44% (*p* = 0.0041)	−71% (*p* < 0.0001)
		20 mg/kg/day Brensocatib	−85% (*p* < 0.0001)	−80% (*p* < 0.0001)	−94% (*p* < 0.0001)
	QD	0.75 mg/kg/day Brensocatib	−9% (*p* = 0.8092)	+2% (*p* = 0.9977)	−1% (*p* = 0.9999)
		3 mg/kg/day Brensocatib	−55% (*p* < 0.0001)	−46% (*p* = 0.0008)	−64% (*p* < 0.0001)
		30 mg/kg/day Brensocatib	−71% (*p* < 0.0001)	−65% (*p* < 0.0001)	−87% (*p* < 0.0001)
*BALB/c*	BID	0.2 mg/kg/day Brensocatib	+26% (*p* = 0.4425)	+13% (*p* = 0.6435)	−1% (*p* = 0.9993)
		2 mg/kg/day Brensocatib	−23% (*p* = 0.5256)	−26% (*p* = 0.1243)	−36% (*p* = 0.0165)
		20 mg/kg/day Brensocatib	−79% (*p* = 0.0008)	−70% (*p* < 0.0001)	−92% (*p* < 0.0001)
	QD	0.3 mg/kg/day Brensocatib	−3% (*p* = 0.9990)	−7% (*p* = 0.9149)	−2% (*p* = 0.9983)
		3 mg/kg/day Brensocatib	−16% (*p* = 0.8297)	−13% (*p* = 0.6769)	−39% (*p* = 0.0058)
		30 mg/kg/day Brensocatib	−46% (*p* = 0.0959)	−52% (*p* = 0.0010)	−84% (*p* < 0.0001)

In addition to comparing brensocatib’s effect within each treatment group, analysis was performed to compare the respective change from vehicle across the two dosing frequencies. Despite the slightly lesser reduction of NSP activities with QD dosing, no statistical difference was observed between similar treatment groups when comparing BID to QD change from vehicle for the three NSP activities (Table 3). As such, the combined BID and QD percent reduction at the highest dose level for C57BL/6 mice were 77%, 71%, and 90% for NE, PR3, and CatG, respectively.

**TABLE 3:** Statistical comparison of BID vs. QD percent change from vehicle at matched dosing levels in C57BL/6 and BALB/c mice. *p*-values derived from Sidak’s multiple comparison test.

		*NE*	*PR3*	*CatG*
***C57BL/6***	Low Dose Brensocatib	0.6557	0.7154	0.2379
	Mid Dose Brensocatib	0.3965	0.9964	0.7873
	High Dose Brensocatib	0.4200	0.4362	0.7433
***BALB/c***	Low Dose Brensocatib	0.4226	0.3244	>0.9999
	Mid Dose Brensocatib	0.9731	0.6802	0.9722
	High Dose Brensocatib	0.2940	0.4308	0.8336

For BALB/c mice, a dose-dependent reduction was observed in bone marrow for all three NSP activities. BID dosing at the highest dose reduced NE, PR3, and CatG by 79% (*p* = 0.0008), 70% (*p* < 0.0001), and 92% (*p* < 0.0001), respectively. QD dosing at the highest dose reduced NE, PR3, and CatG by 46% (*p* = 0.0959), 52% (*p* = 0.0010), and 84% (*p* < 0.0001), respectively (Table 2). Unlike for PR3 and CatG, the reduction of NE activity was not statistically significant for QD dosing, which may indicate that the brensocatib QD dosing frequency may be insufficient to robustly reduce NE activity in BALB/c mice, at least within the 7-day time frame. However, it is important to note that the animals that displayed a lesser reduction in NE/NSP reduction were the same animals exhibiting barbering/aggression. This behavior may have caused undue stress to the animals and interfered with the drug’s effect, resulting in a less robust reduction of NSP activities.

In addition to comparing brensocatib’s effect within each treatment group, analysis was performed to compare the respective change from vehicle across the two dosing frequencies. No statistical difference was observed between similar treatment groups (Table 3). The combined BID and QD percentage reductions at the highest dose level for BALB/c mice were 62%, 61%, and 88% for NE, PR3, and CatG, respectively.

#### 3.1.3 PK: Sprague Dawley and Wistar rats

**FIGURE 3:**
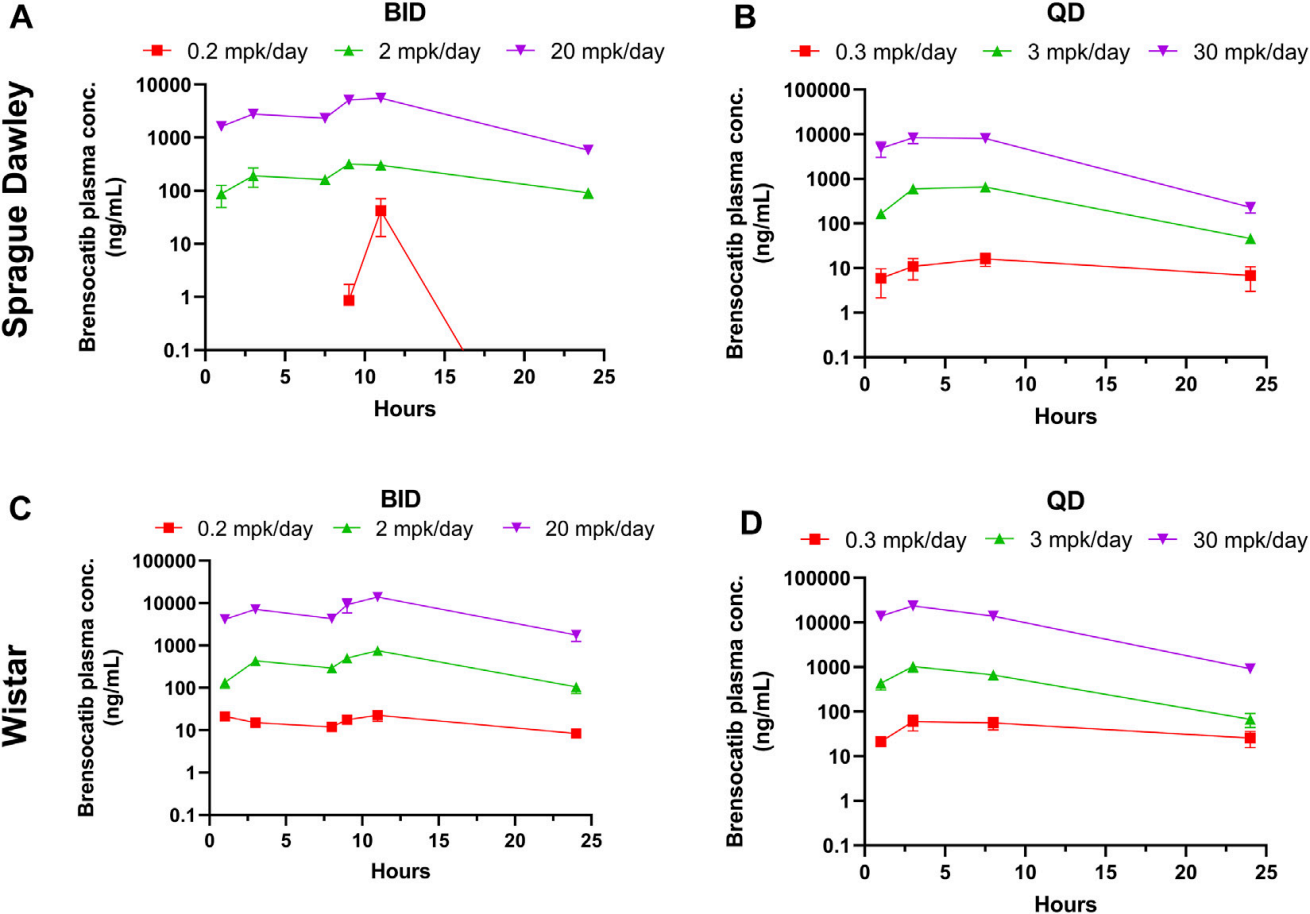
Bensocatib Plasma Concentration from Repeated Dose Administration of Brensocatib by Oral Gavage. **(A)** BID in Sprague Dawley (SD) rats, **(B)** QD in SD rats, **(C)** BID in Wistar rats, **(D)** QD in Wistar rats. Data are plotted as mean ± SEM (*n* = 4, except for 0.2 mpk/day BID for SD at 1, 3, 9, and 24 h where *n* = 3; 2 mpk/day BID at 11 h for SD where *n* = 3; 0.3 mpk/day QD at 3 h for SD where *n* = 3; 3 mpk/day BID for SD at 3 and 24 h where *n* = 3; 30 mpk/day BID at 7.5 h for SD where *n* = 3; 0.2 mpk/day BID at 16 h for Wistar where *n* = 3; and 30 mpk/day QD at 8 h for Wistar where *n* = 3). mpk/day = mg/kg/day.

Brensocatib was administered orally to male and female Sprague Dawley rats for 9 consecutive days either BID or QD. The brensocatib plasma levels over the course of the first 24 h of drug exposure are shown in Figure 3, and the PK parameters are listed in Table 4.

**TABLE 4:** PK parameters of brensocatib in Sprague Dawley and Wistar rats during the first 24 h of BID or QD dosing.

		BID			QD		
SD	Dose (mg/kg/day)	0.2	2	20	0.3	3	30
	AUC_0–24h_ (ng•h/mL)	124 ± 81	4728 ± 421	72,568 ± 3401	269 ± 128	11,098 ± 107	104,278 ± 25,039
	AUC_(0–24)_ (µmol•h/L)	0.29 ± 0.19	11.24 ± 1.00	172.57 ± 8.09	0.64 ± 0.30	26.39 ± 0.25	247.99 ± 59.54
	t_1/2_ (h), AM dose	n/a	13.49 ± 9.16	14.10 ± 1.55	5.36 ± 2.38	4.46 ± 0.37	3.88 ± 0.04
	t_1/2_ (h), PM dose	0.55 ± 0.02	7.42 ± 1.16	4.03 ± 0.44	n/a	n/a	n/a
Wistar	Dose (mg/kg/day)	0.2	2	20	0.3	3	30
	AUC_0–24h_ (ng•h/mL)	377 ± 78	9817 ± 1815	173,277 ± 37,673	1036 ± 291	11,720 ± 2327	266,484 ± 22,039
	AUC_(0–24)_ (µmol•h/L)	0.90 ± 0.18	23.35 ± 4.32	412.07 ± 89.59	2.46 ± 0.69	27.87 ± 5.53	633.73 ± 52.41
	t_1/2_ (h), AM dose	5.48 ± 1.76	10.16 ± 1.47	7.81 ± 1.38	34.31 ± 20.32	5.14 ± 0.50	4.41 ± 0.18
	t_1/2_ (h), PM dose	6.45 ± 0.55	4.99 ± 0.33	4.33 ± 0.16	n/a	n/a	n/a

AUC, area under the concentration-time curve; t_1/2_ = half life. Data are presented as mean ± SEM (n = 4, except for SD, 0.2 mg/kg/day AUC, where n = 2 due to confirmed PK, outliers by Grubbs test; SD, 2 mg/kg/day AM t_1/2_ where n = 2 due to inability to calculate parameter, SD, 2 mg/kg/day AM t_1/2_ where n = 3 due to confirmed outlier by Grubbs test, SD, 0.3 mg/kg/day AUC, where n = 3 due to confirmed PK, outlier by Grubbs test; SD, 3 mg/kg/day AUC, where n = 2 due to confirmed PK, outliers by Grubbs test; SD, 0.3 mg/kg/day AM t_1/2_ where n = 3 due to confirmed outlier by Grubbs test, and Wistar 0.2 mg/kg/day where n = 3 due to confirmed PK, outliner by Grubbs test). n/a, not applicable or could not be calculated.

Both dosing regimens showed a dose-dependent trend of plasma exposure (Figure 3). The plasma AUC_0–24h_ for BID dosed rats was 0.29, 11.24, and 172.57 μmol h/L for the 0.2, 2, and 20 mg/kg/day doses, respectively. The plasma AUC_0–24h_ in QD dosed rats was 0.64, 26.39, and 247.99 μmol h/L for the 0.3, 3, and 30 mg/kg/day doses, respectively (Table 4). A ratio of QD to BID AUC ranged from 1.4 to 2.3 for the respective doses.

The t_1/2_ for BID dosing for the morning dose was 13.49 and 14.10 h for the 2 and 20 mg/kg/day doses, respectively. Due to plasma concentrations being below the limit of detection for the 0.2 mg/kg/day group, no calculation was performed. The BID evening t_1/2_ was 0.55, 7.42, and 4.03 h for the three doses. The t_1/2_ for QD dosing was 5.36, 4.46, and 3.88 h for the 0.3, 3, and 30 mg/kg/day doses, respectively (Table 4). Note that both dosing regimens showed large variability in the calculated half-life of brensocatib, which may be due to a large number of data outliers that were excluded from the dataset and limited the ability to calculate certain PK parameters.

A similar study was repeated in male and female Wistar rats but with 7 days of dosing instead of 9 days. The brensocatib plasma levels over the course of the first 24 h of drug exposure are shown in Figure 3, and the PK parameters are listed in Table 4. Both dosing regimens showed a dose-dependent trend of plasma exposure (Figure 3). The plasma AUC_0–24h_ in BID dosed rats was 0.90, 23.35, and 412.07 μmol h/L for the 0.2, 2, and 20 mg/kg/day doses, respectively, while the plasma AUC_0–24h_ in QD dosed rats was 2.46, 27.87, and 633.73 μmol h/L for the 0.3, 3, and 30 mg/kg/day doses, respectively (Table 4). A 1.2- to 2.7-fold difference between the QD AUC and BID AUC for the respective doses were observed due to a greater daily dose for the QD groups.

The t_1/2_ for BID dosing was 5.48, 10.16, and 7.81 h for the 0.2, 2, and 20 mg/kg/day doses, respectively, in the morning and were comparable to the evening dose t_1/2_, which was 6.45, 4.99, and 4.33 h, respectively. The t_1/2_ for QD dosing was 34.31, 5.14, and 4.41 h for the 0.3, 3, and 30 mg/kg/day doses, respectively (Table 4). Overall, both dosing regimens showed a relatively short half-life of brensocatib.

#### 3.1.4 PD: Sprague Dawley and Wistar rats

**FIGURE 4:**
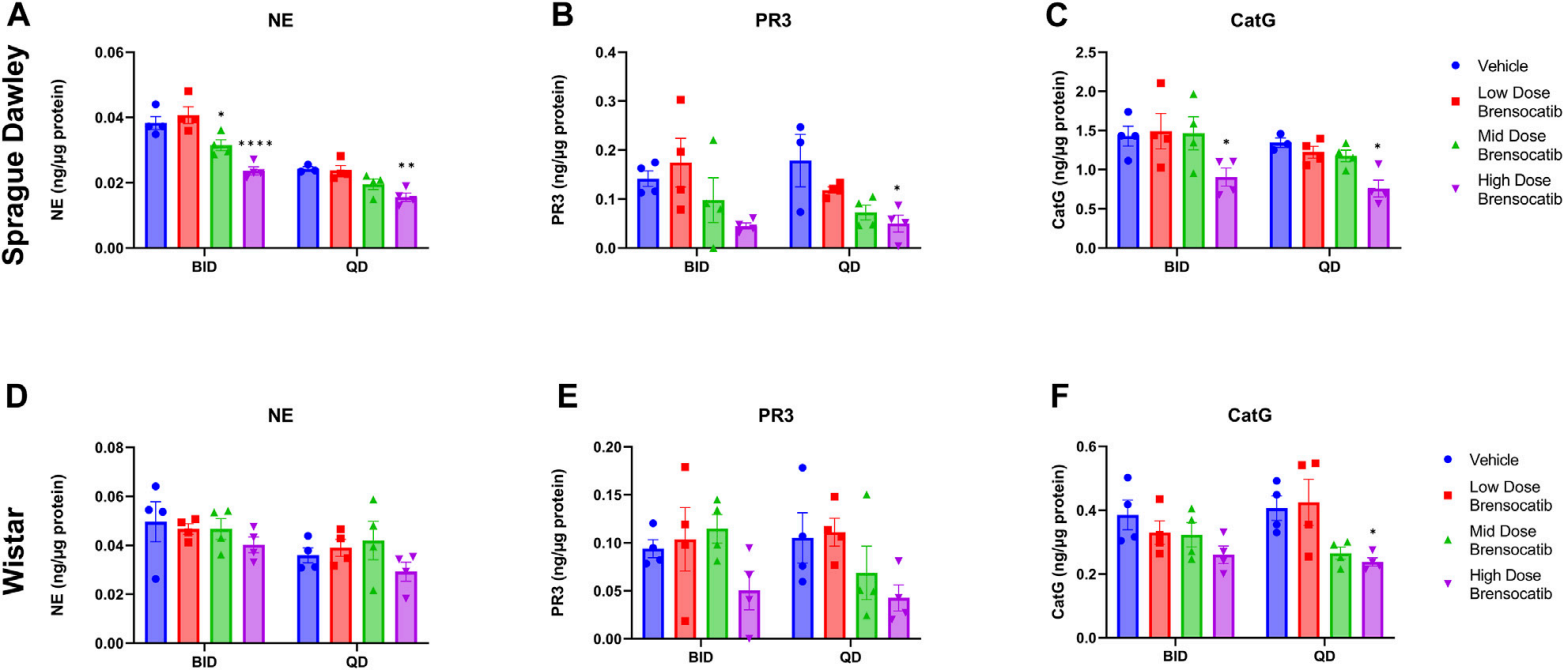
NSP Activities in Bone Marrow of Sprague Dawley **(A–C)** and **(D–F)** Wistar Rats Administered Repeated Doses of Brensocatib. Data are plotted as mean ± SEM (*n* = 4, except for SD NE QD vehicle, SD PR3 QD vehicle, and SD CatG QD vehicle where *n* = 3). *, *p* < 0.05 vs. vehicle.

The bone marrow NSP activity levels after the 9- or 7-day repeated doses of brensocatib in Sprague Dawley and Wistar rats, respectively, are shown in Figure 4 for both the BID and QD dosing regimens.

For Sprague Dawley rats, a dose-dependent reduction was observed in the bone marrow NSP activity levels for all three NSPs. BID dosing at the highest dose reduced NE, PR3, and CatG by 38% (*p* < 0.0001), 68% (*p* = 0.0907), and 37% (*p* = 0.0420), respectively. QD dosing at the highest dose reduced NE, PR3, and CatG by 36% (*p* = 0.0078), 72% (*p* = 0.0285), and 43% (*p* = 0.0348), respectively (Table 5).

**TABLE 5:** Brensocatib treatment percentage change from vehicle control for BID and QD dosing in Sprague Dawley and Wistar rats. *p*-values derived from Sidak’s multiple comparison test.

			*Change From Vehicle Control (%)*		
			NE	PR3	CatG
*Sprague Dawley*	BID	0.2 mg/kg/day Brensocatib	+6% (*p* = 0.6846)	+23% (*p* = 0.8292)	+4% (*p* = 0.9857)
		2 mg/kg/day Brensocatib	−18% (*p* = 0.0243)	−31% (*p* = 0.6727)	+2% (*p* = 0.9972)
		20 mg/kg/day Brensocatib	−38% (*p* < 0.0001)	−68% (*p* = 0.0907)	−37% (*p* = 0.0420)
	QD	0.3 mg/kg/day Brensocatib	−2% (*p* = 0.9973)	−34% (*p* = 0.4869)	−9% (*p* = 0.9235)
		3 mg/kg/day Brensocatib	−19% (*p* = 0.2196)	−59% (*p* = 0.0837)	−13% (*p* = 0.8203)
		30 mg/kg/day Brensocatib	−36% (*p* = 0.0078)	−72% (*p* = 0.0285)	−43% (*p* = 0.0348)
*Wistar*	BID	0.2 mg/kg/day Brensocatib	−6% (*p* = 0.9661)	+10% (*p* = 0.9848)	−14% (*p* = 0.7146)
		2 mg/kg/day Brensocatib	−6% (*p* = 0.9676)	+22% (*p* = 0.8768)	−16% (*p* = 0.6392)
		20 mg/kg/day Brensocatib	−19% (*p* = 0.4767)	−46% (*p* = 0.4135)	−32% (*p* = 0.1106)
	QD	0.3 mg/kg/day Brensocatib	+8% (*p* = 0.9636)	+6% (*p* = 0.9963)	+5% (*p* = 0.9843)
		3 mg/kg/day Brensocatib	+17% (*p* = 0.7823)	−35% (*p* = 0.5635)	−35% (*p* = 0.0605)
		30 mg/kg/day Brensocatib	−19% (*p* = 0.7283)	−59% (*p* = 0.1437)	−41% (*p* = 0.0202)

When comparing the respective change from vehicle across the two dosing frequencies, there was no statistical difference observed between BID and QD dosing at each dosing level (Table 6). The combined BID and QD percent reduction at the highest dose level for Sprague Dawley rats was 37%, 70%, and 40% for NE, PR3, and CatG, respectively.

**TABLE 6:** Statistical comparison of BID vs. QD percentage change from vehicle at matched dosing levels in Sprague Dawley and Wistar rats. *p*-values derived from Sidak’s multiple comparison test.

		*NE*	*PR3*	*CatG*
***SD***	Low Dose Brensocatib	0.6794	0.1760	0.7615
	Mid Dose Brensocatib	0.9967	0.6993	0.6889
	High Dose Brensocatib	0.9808	0.9989	0.9585
***Wistar***	Low Dose Brensocatib	0.7801	0.9986	0.4786
	Mid Dose Brensocatib	0.4620	0.2491	0.4881
	High Dose Brensocatib	>0.9999	0.9692	0.8924

For Wistar rats, BID dosing at the highest dose reduced NE, PR3, and CatG by 19% (*p* = 0.4767), 46% (*p* = 0.4135), and 32% (*p* = 0.1106), respectively. QD dosing at the highest dose reduced NE, PR3, and CatG by 19% (*p* = 0.7283), 59% (*p* = 0.1437), and 41% (*p* = 0.0202), respectively (Table 5). In addition to comparing brensocatib’s effect within each treatment group to their respective vehicle controls, analysis was performed comparing the respective change from vehicle across the two dosing frequencies. No statistical difference was observed between similar treatment groups (Table 6). The combined BID and QD percent reduction at the highest dose level for Wistar rats were 19%, 53%, and 37% for NE, PR3, and CatG, respectively.

### 3.2 Onset and recovery

**FIGURE 5:**
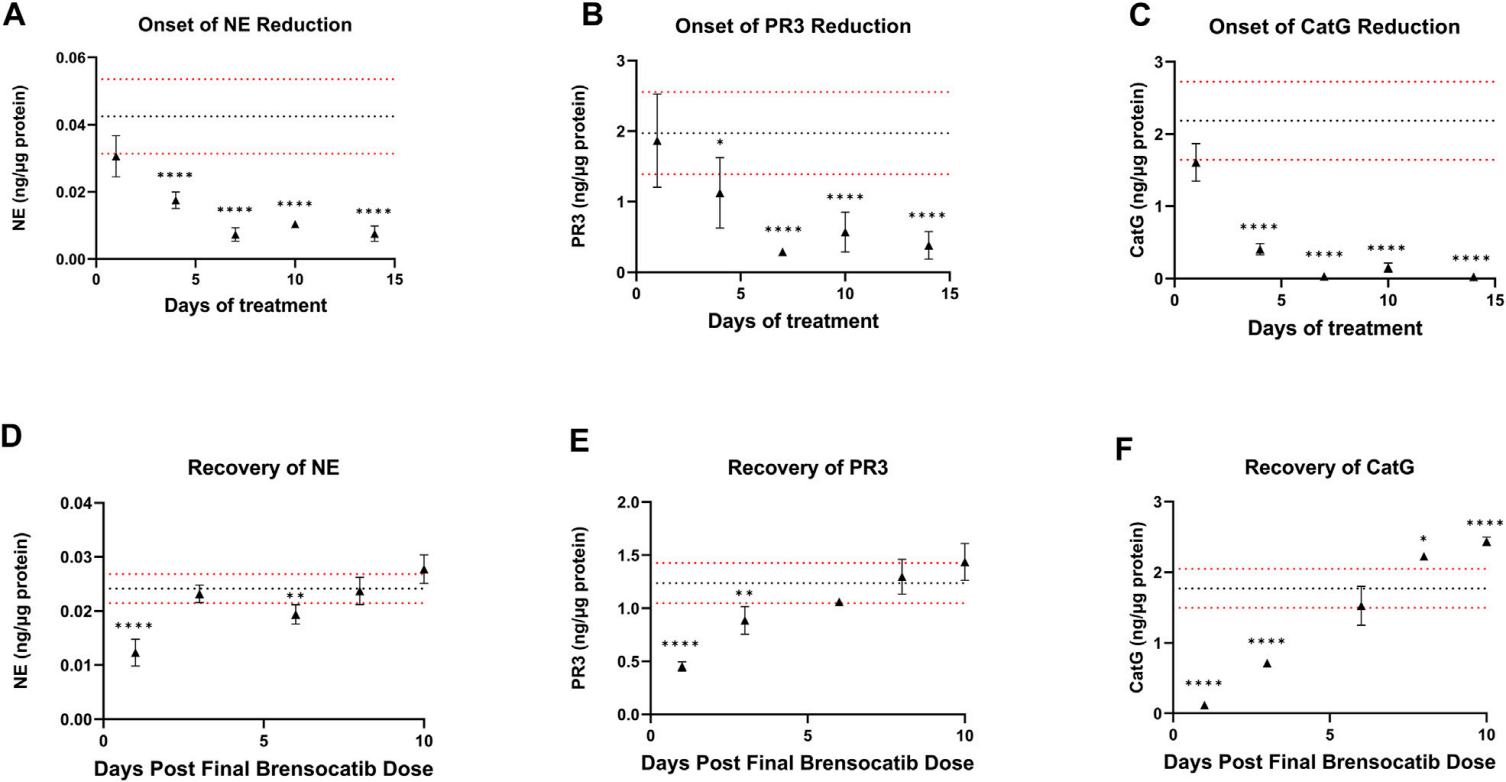
**(A–C)** Onset and **(D–F)** Recovery of NSP Activities in Bone Marrow of C57BL/6 Mice Administered Repeated Doses of Brensocatib. The dotted horizontal lines represent the average (black) ± 1 SD (red) for the vehicle control. Data are plotted as mean ± SD (*n* = 4 for all brensocatib treatment groups, except for onset NE day 10, recovery PR3 day 6, and recovery CatG day 8 where *n* = 3 after removal of an outlier determined by Grubb’s test; *n* = 20 for vehicle control, except onset PR3 and recovery CatG where *n* = 19 after removal of outlier determined by Grubb’s test). *, *p* < 0.05 vs. vehicle; **, *p* < 0.01 vs. vehicle; ***, *p* < 0.001 vs. vehicle; ****, *p* < 0.0001 vs. vehicle.

For the Onset and Recovery investigations, a dose of 10 mg/kg/day was used as it falls approximately halfway between the mid and high doses tested in the above studies, which both showed robust reduction of NSP activities. Plasma was collected for PK analysis during the onset and recovery studies. Consistent Cmax, AUC, and t_1/2_ were observed in those studies (data not shown). The bone marrow NSP activity levels after 1-, 4-, 7-, 10-, and 14-day repeated doses of brensocatib, and 1-, 3-, 6-, 8-, and 10- days post final dose are shown in Figure 5.

During the course of the onset study, a gradual duration-dependent reduction was observed in bone marrow NSP levels for all three NSPs that reached a maximum after approximately 7 days (∼8-day with modeling) with no further attenuation through day 14 (Figures 5A–C; Table 7; Table 9). When modeled with the min and max free for the curve fit using the percent remaining NSP activity, it took 4.3, 4.5, and 2.8 days to reach a 50% reduction in NE, PR3, and CatG, respectively. The maximum percent reduction as determined using the same model fitting was 80%, 76%, and 97% for NE, PR3, and CatG, respectively, which took 6.4, 8.0, and 8.1 days, respectively (Table 9).

**TABLE 7:** NSP percentage change from vehicle control after 1, 4, 7, 10, and 14 days of brensocatib 5 mg/kg BID treatment in C57BL/6 mice. *p*-values derived from Dunnett’s multiple comparison test.

	Percent change from vehicle		
	NE	PR3	CatG
**1-day**	−28% (*p* = 0.0826)	−5% (*p* = 0.9972)	−26% (0.0727)
**4-day**	−59% (*p* < 0.0001)	−43% (*p* = 0.0236)	−81% (*p* < 0.0001)
**7-day**	−83% (*p* < 0.0001)	−85% (*p* < 0.0001)	−99% (*p* < 0.0001)
**10-day**	−76% (*p* < 0.0001)	−71% (*p* < 0.0001)	−93% (*p* < 0.0001)
**14-day**	−82% (*p* < 0.0001)	−81% (*p* < 0.0001)	−99% (*p* < 0.0001)

The recovery of the bone marrow NSP activities was monitored after the last dose of their 8-day repeated brensocatib treatment (Figures 5D–F). When compared to the appropriate vehicle control, maximum reduction in NSP activity was observed 1 day post final dose, which was fully recovered visually by approximately 6–8 days post final dose for all NSPs (Figures 5D–F; Table 8). A similar trend was observed when modeled using a Free Min and Max Boltzmann Curve. In this case, the estimated recovery times for NE, PR3, and CatG were 6.6, 7.5, and 5.8 days, respectively (Table 9).

**TABLE 8:** NSP percentage change from vehicle control after C57BL/6 mice were allowed to recover from 8-days of brensocatib treatment for 1-, 3-, 6-, 8-, and 10-days. *p*-values derived from Dunnett’s multiple comparison test.

	Percent change from vehicle		
	NE	PR3	CatG
**1-day**	−49% (*p* < 0.0001)	−63% (*p* < 0.0001)	−93% (*p* < 0.0001)
**3-day**	−4% (*p* = 0.9544)	−28% (*p* = 0.0024)	−60% (*p* < 0.0001)
**6-day**	−20% (*p* = 0.0067)	−14% (*p* = 0.3753)	−14% (*p* = 0.2431)
**8-day**	−2% (*p* = 0.9986)	+5% (*p* = 0.9691)	+26% (*p* = 0.0133)
**10-day**	+15% (*p* = 0.0607)	+16% (*p* = 0.1549)	+38% (*p* < 0.0001)

**TABLE 9:** Trend of NSP reduction after 14 days of brensocatib treatment & trend of NSP recovery after 8 days of brensocatib treatment—free min and max for curve fit.

	*Onset*			*Recovery*			
	Time to reach 50% reduction (Days)	Maximum reduction (plateau) (%)	Time to reach max reduction (Days)	Time to reach 50% recovery (Days)	Time to reach 90% recovery (Days)	Time to reach 95% recovery (Days)	Time to reach 100% recovery (Days)
*NE*	4.3	80	6.4	0.8	4.9	5.6	6.6
*PR3*	4.5	76	8.0	1.4	5.2	6.2	7.5
*CatG*	2.8	97	8.1	3.3	5.2	5.5	5.8

Other modeling methods are available and may result in different estimations of time to onset and time to recovery; however, maximum reduction in NSPs was visually observed after ∼7 days and recoveries were nearly symmetrical.

## 4 Discussion

Based on this investigation of brensocatib in the various species and strains, it is now clear that species, more so than strains, influence the PK and PD of the drug. More specifically, rats displayed a longer plasma half-life than mice. Despite this, more robust PD effects were observed in mice, with greater reductions in NE, PR3, and CatG at the equivalent dose. Furthermore, the order of PD effect on the three NSPs was different between the two species, but was consistent between both strains of the same species. While the species interacted with the PD effect, the frequency of dosing did not affect the level of NSP reduction when the QD dose was 1.5 times the BID daily dose. Maximum reduction in NSP activity was observed after ∼7 days and recoveries to baseline NSP levels were nearly symmetrical. These results can therefore be translated to evidence-based study design practices for future *in vivo* investigations, particularly with disease models that may be available in both rodent species. The importance of such studies stems from the known role of NSPs in innate immune functions and, when dysregulated, in disease.

NSPs play an important role in innate immune functions related to the neutrophil, contributing to inflammatory regulation and modulation and pathogen destruction (Pham, 2006; Pham, 2008; Stapels et al., 2015). Of concern in chronic inflammatory diseases, neutrophil accumulation and dysregulated activation can result in excessive secretion of active NSPs, causing damaging inflammation and tissue matrix destruction. Moreover, elevated NSPs are associated with increased disease severity and morbidity in such neutrophilic diseases as chronic obstructive pulmonary disease, bronchiectasis, cystic fibrosis, and others (Almansa et al., 2012; Chalmers et al., 2017; Dai et al., 2017; Dittrich et al., 2018; Fresneda Alarcon et al., 2021). Given NSP involvement in diverse pathologies and correlation with disease severity, DPP1 inhibitors are being pursued as pharmaceutical targets (Laine and Busch-Petersen, 2010; Hou et al., 2019; Chalmers et al., 2020).

A particular challenge with the development of DPP1 inhibitors targeting NSPs in inflammatory disease is the recognition that DPP1 activates NSPs during granulocyte development, which takes place in the bone marrow (Korkmaz et al., 2010). Therefore, one must consider the ability of a DPP1 inhibitor to reach the bone marrow and the requirement for sustained exposure to effectively reduce NSP activities (Methot et al., 2007). While this may seem trivial, previous DPP1 inhibitors have failed to show efficacy in the clinic presumably due to lack of, or insufficient, NSP reduction (Miller et al., 2017) despite preclinical and clinical evidence of robust DPP1 inhibition in both mice and human (Laine et al., 2011; Miller et al., 2017). Perhaps tellingly, one study only reported measurements of DPP1 inhibition in mouse plasma and lung homogenates, without investigating the bone marrow (Laine et al., 2011). Another important consideration is the time needed to achieve a full therapeutic effect and a return to baseline levels following termination of treatment; both of these processes should follow the timeline of neutrophil maturation, which may differ among species (Laine et al., 2011; Gardiner et al., 2016). While some of these investigations can be answered *in vitro* by differentiating hematopoietic stem cells in the presence of DPP1 inhibitors (Zhang et al., 2019), *in vivo* models are currently the most appropriate systems to assess a DPP1 inhibitor’s ability to reach the target tissue (i.e., the bone marrow), reduce NSP activities, characterize its pharmacokinetics, and recover NSP activity post treatment.

In the rodent species and strains tested herein with brensocatib treatment, we observed dose-dependent PK exposure responses (AUC and Cmax) with a short half-life of approximately 2.5 h for mice and 8.0 h for rats. The variations in the estimates of the half-lives across the BID and QD dosing regimens are attributed to this being a sparse PK analysis and more precise estimates would likely be observed in a rich PK analysis with more frequent time points and a greater number of animals. However, due to in-life PK sample volume constraints as defined by IACUC, only sparse sampling could be conducted when running these joint, multiday PK/PD studies. Furthermore, both mice and rats dosed QD had equivalent NSP reductions compared to BID dosing when the QD dose was 1.5 times the BID daily dose. NE, PR3, and CatG activities were reduced by approximately 77%, 71%, and 90% in C57BL/6 mice, 62%, 61%, and 88% in BALB/c mice, 37%, 70%, and 40% in SD rats, and 19%, 53%, and 37% in Wistar rats, respectively, at 20/30 (BID/QD) mg/kg/day brensocatib. Maximum reduction in NSP activity was observed after ∼7 days and recoveries to baseline NSP levels were nearly symmetrical. This data matches very closely with a different study investigating the effect of DPP1 inhibition on NSP activities (Gardiner et al., 2016).

DPP1 sequences are known to vary slightly across species so we hypothesized that the greater reduction in NSP activities in mice compared to rats may be due to a higher binding affinity of brensocatib to the DPP1 active site in mice compared to rats; however, this will need to be verified in future studies. For both species, the differences in the extent of reductions among the NSP activities may be explained by differences in the binding affinity of each NSP zymogen to the DPP1 binding pocket. The higher the binding affinity of the NSP zymogen to the DPP1 binding pocket, the greater the likelihood that the inactive NSP zymogen will be cleaved to its active form relative to the other NSP zymogens, in the presence of a competitive inhibitor like brensocatib. Our results suggest that the binding affinities for mouse DPP1 is strongest to the zymogens of PR3 followed by NE and then CatG; in contrast, the binding affinity of rat DPP1 may be strongest to NE followed by CatG and then PR3. Future work may benefit from quantifying this parameter. In addition to differences in binding affinity, another possible explanation for the greater NSP reduction observed in mice is related to drug exposure at the target tissue. For NSPs to be effectively reduced, the drug must distribute to, and persist in, the bone marrow compartment. In this study, PK plasma exposure was evaluated, but future studies may benefit from measuring the drug concentration in the bone marrow, where DPP1 cleaves the NSPs into their active forms. This measurement may better inform on the distribution profile and half-life of the drug in the active site compartment.

Slightly lesser reductions in NSP activities were also observed in animals that exhibited barbering and/or aggression, which was exclusively observed in some BALB/c males. Behaviorally, it has been reported that BALB/c mice are more anxious, less social and, particularly for males, more natively aggressive compared to C57BL/6 mice, which rarely display aggressive behaviors (Nicholson et al., 2009; An et al., 2011; Heinla et al., 2018). These behaviors may explain why a slightly lesser reduction in NSP activities was observed only for those animals, since these behaviors are related to, or induce, high stress, which may increase drug metabolism and reduce brensocatib’s inhibitory effect (Panagiotis Kourounakis and Tsiakitzis, 2010; Konstandi et al., 2014). Unfortunately, separation of mice displaying these behaviors may still cause distress as they are social, not solitary, animals (Nicholson et al., 2009; Hickman et al., 2017). Therefore, when designing a study, either naïve or indication based, choosing a species, strain, and sex that is docile may optimize compound investigations *in vivo* (Hickman et al., 2017). Other methods to regulate animal stress that may confound study results should also be considered such as proper group housing, environmental enrichment, and minimizing unnecessary handling and traffic through the animal room (Animals, 2008).

Overall, these results will facilitate future *in vivo* study dosing considerations with brensocatib treatment, such as the timing of prophylactic or therapeutic administration, the choice of species, strain, and sex, and the dosage and dosing frequency. For studies where QD dosing is preferred, either to reduce animal handling or stress, both which may confound results and/or reduce study costs, choosing a QD dose that is 1.5-times the desired BID dose may be sufficient to achieve equivalent NSP activity reductions. Furthermore, for indication studies that require or may benefit from maximum reduction in NSPs, brensocatib should be administered for at least 7 days prior to disease induction to achieve an optimal PD effect.

## Data Availability

The original contributions presented in the study are included in the article/Supplementary Material, further inquiries can be directed to the corresponding author.
